# The complete mitochondrial genome of *Stegobium paniceum* (Coleoptera: Anobiidae)

**DOI:** 10.1080/23802359.2016.1241679

**Published:** 2016-11-12

**Authors:** Wen-Jia Yang, Xing-Ying Cai, Kang-Kang Xu, Yu Cao, Yong-Lu Meng, Can Li

**Affiliations:** Key & Special Laboratory of Guizhou Education Department for Pest Control and Resource Utilization, College of Biology and Environmental Engineering, Guiyang University, Guiyang, China

**Keywords:** *Stegobium paniceum*, drugstore beetle, mitochondrial genome

## Abstract

The complete mitochondrial genome of *Stegobium paniceum* (Coleoptera: Anobiidae) is a circular DNA molecule of 15,271 bp (GenBank accession number XK819317), and its nucleotide composition is biased towards A + T nucleotides (78.32%). This genome comprises 13 protein-coding genes, 2 ribosomal RNA genes, 22 transfer RNA (tRNA) genes, and an A + T-rich region. The gene order of *S. paniceum* was similar to those found in other known Coleoptera species. Sixteen reading frame overlaps and six intergenic regions were found in the mitochondrial genome of *S. paniceum*. All 22 tRNA genes have the typical cloverleaf secondary structure, with an exception for *trnS_1_* (AGN). The phylogenetic relationships based on neighbour-joining method revealed that *S. paniceum* is closely related to *Apatides fortis*, which is consistent with the traditional morphological classification.

The drugstore beetle, *Stegobium paniceum* (Linnaeus) (Coleoptera: Anobiidae), is a major pest that cause tremendous damage to stored grains and seeds, packaged food products, animal-, and plant-derived products, including more than 300 drug materials (Li et al. 2009; Abdelghany et al. 2010). In this study, the *S. paniceum* samples were collected from medicinal materials company in Guizhou province of China (N26°31′, E106°43′). The specimen was deposited in Insect Collection (Accession Number GYU-Col-20090001-2), College of Biology and Environmental Engineering, Guiyang University, Guiyang, China.

The complete mitochondrial genome of *S. paniceum* (GenBank accession number XK819317) has been sequenced and annotated. It is a typical circular DNA molecule of 15,271 bp in length, larger than *Eucryptorhynchus brandti* (15,122 bp) and smaller than *Tribolium castaneum* (15,883bp), *Dastarcus helophoroides* (15,878 bp), and *Hycleus chodschenticus* (16,257 bp) (Liu et al. 2014; Zhang et al., 2014; Nan et al. 2016; Yuan et al. 2016).The nucleotide composition of mitochondrial genome of *S. paniceum* is heavily biased towards A + T nucleotides, accounting for A (41.62%), T (36.70%), G (9.06%), and C (12.62%). The AT-skew and GC-skew of this genome were 0.063 and −0.164, respectively.

The mitochondrial genome of *S. paniceum* contains 37 genes, including13 protein-coding genes (PCGs), 2 ribosomal RNA (rRNA) genes, and 22 transfer RNA (tRNA) genes. The gene order of *S. paniceum* was similar to those found in other known Coleoptera species. Gene overlaps 39 bp that have been found at 16 gene junctions, the longest 8 bp overlapping exists between *trnW* and *trnC*. The *S. paniceum* mitochondrial genome harbours six intergenic regions (*trnS_2_* and *nad1* share 20 nucleotides, *trnM* and *nad2* share three nucleotides, *nad4L* and *trnT* share two nucleotide, *trnY and cox1* share a nucleotide, *trnP* and *nad6* share a nucleotide, and *nad1* and *trnL_1_* share a nucleotide). With an exception for *trnS_1_* (AGN), all tRNAs have the typical cloverleaf secondary structure, which are common in most animal mitochondrial genome (Wolstenholme 1992). The length of these tRNAs vary from 62 bp for *trnI*, *trnL1, trnY* to 70 bp for *trnM*, A + T content ranged from 70.31% (*trnF*) to 90.48% (*trnE*).

Two rRNA genes (*rrnL* and *rrnS*) are located between *trnL_1_* and *trnV*, and between *trnV* and the A + T-rich region, respectively. The size of *rrnL* and *rrnS* of *S. paniceum* is 1254 bp and 755 bp long, respectively. Of the 13 PCGs, 11 genes start with ATN codons, including five ATAs (*cox2*, *nad3*, *nad4*, *nad6*, and *nad2*), four ATGs (*atp6*, *cox3*, *nad4L*, and *cob*), two ATTs (*atp8* and *nad5*). However, *cox1* and *nad1* used AAA and TTG as start codon, respectively. Eight PCGs terminate with the conventional stop codons TAA (*atp6*, *atp8*, *nad4L*, *nad6*, and *nad2*) or TAG (*nad3*, *cob*, and *nad1*), the remaining PCGs including *cox1*, *cox2*, *cox3*, *nad4*, and *nad5* use a single T as stop codon. The major A + T-rich region was located between *rrnS* and *trnI* genes with a length of 795 bp long, and the A + T content was 90.69%. Based on the concatenated amino acid sequences of 13 PCGs, the neighbour-joining method was used to construct the phylogenetic relationship of *S. paniceum* with 13 other representative beetles. The mitochondrial genome of neuropteran *Ascalohybris subjacens* (NC_011277) was used as an outgroup. The results demonstrated that *S. paniceum* is more closely related to *Apatides fortis* than other beetles (Figure 1), which is consistent with the traditional morphological classification.

**Figure 1.**
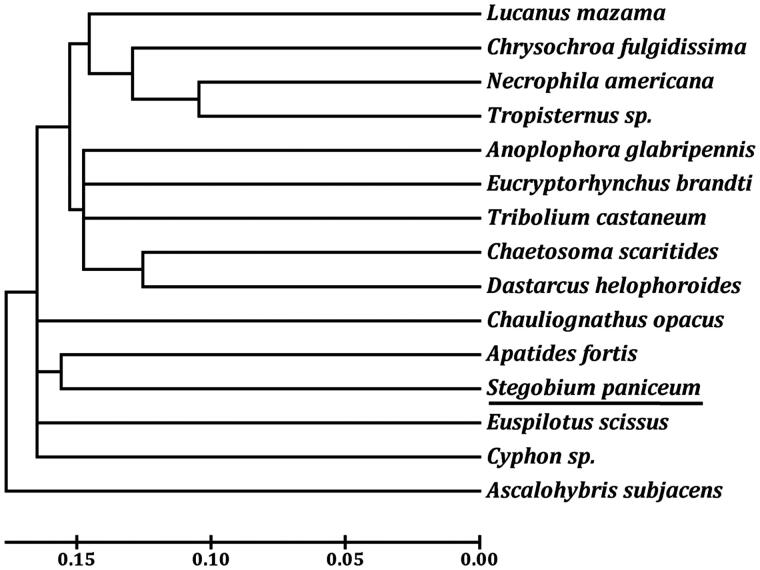
The neighbour-joining phylogenetic tree of *S. paniceum* and other beetles was constructed based on 13 mitochondrial protein-coding genes. The GenBank accession numbers used for tree constructed are as follows: *A. fortis* (NC_013582), *Anoplophora glabripennis* (NC_008221), *Chaetosoma scaritides* (NC_011324), *Chrysochroa fulgidissima* (NC_012765), *Chauliognathus opacus* (NC_013576), *Cyphon* sp. (NC_011320), *D. helophoroides* (NC_024271), *E. brandti* (NC_025945), *Euspilotus scissus* (NC_018353), *Lucanus mazama* (NC_80 013578), *Necrophila americana* (NC_018352), *T. castaneum* (NC_003081), and *Tropisternus* sp. (NC_018349).
